# Lemierre's syndrome - A new causative organism and life-threatening complication

**DOI:** 10.1186/1749-8090-10-S1-A362

**Published:** 2015-12-16

**Authors:** Mira Pecheva, Alexandra Fielding, Jakub Kadlec, Russell Phillips, Filip Van Tornout

**Affiliations:** 1 Department of Thoracic Surgery, Norfolk and Norwich University Hospital, Colney Ln, Norwich NR4 7UY, UK; 2 Department of Respiratory Medicine, Norfolk and Norwich University Hospital, Colney Ln, Norwich NR4 7UY, UK

## Background/Introduction

Lemierre's syndrome is classically caused by an acute oropharyngeal infection, with subsequent Internal Jugular Vein (IJV) thrombosis and dissemination to secondary sites, with Fusobacterium necrophorum as the main causative organism. We present the case of a 43 year old woman with pharyngodynia, rigors and shortness of breath (SOB). Computer tomography (CT) revealed a bilateral tonsillar bed infection, thrombosis of the right IJV, with positive blood cultures for Slackia species, a gram-positive anaerobic bacterium previously isolated from human faeces. The patient also had bilateral cavitating lung nodules and empyema, requiring pleural decortication on the left. The case was complicated by haemoptysis and a right sided pulmonary artery aneurysm, thought to be mycotic in nature and radiologically embolised. The patient made a full clinical recovery, with complete resolution of the aneurysm and cavitating nodules on final imaging.

## Aims/Objectives

This case highlights the classic clinical features of Lemierre's syndrome and identifies a new causative organism. It also recognises the potentially fatal complication of mycotic aneurysm formation.

## Method

CT imaging demonstrated bilateral gas locules in the tonsillar beds, right IJV thrombosis and bilateral cavitating lung nodules in keeping with septic emboli. The patient also had bilateral empyema, requiring left sided pleural decortication and lung abscess marsupialisation via Video-Assisted Thoracoscopy. A further CT was performed following frank haemoptysis and showed an aneurysm of the right upper pulmonary artery, which was radiologically embolised.

## Results

Blood cultures were positive for Slackia species. Pneumococcal, aspergillus, hepatitis and autoimmune antibody screens were negative. Complement and immunoglobulin titres were normal. Follow up CT at 6 months revealed complete resolution of the pulmonary artery aneurysm and cavitating nodules.

## Discussion/Conclusion

The patient presented with the classic features of Lemierre's syndrome, namely oropharyngeal infection, thrombosis of the IJV and septic emboli. Causative organisms include Fusobacterium necrophorum, Streptococci and Staphylococcus. The organism in this case, of the genus Slackia, is an obligate Gram positive anaerobe not previously associated with Lemierre's syndrome. Furthermore, Lemierre's syndrome has been associated with intracranial and carotid artery aneurysms. Here we describe mycotic aneurysm formation distal to the head and neck, which fully resolved.

## Consent

Written informed consent was obtained from the patient for publication of this abstract and any accompanying images. A copy of the written consent is available for review by the Editor of this journal.

